# Urothelial carcinoma following kidney transplantation: a narrative review of Chinese insights and challenges from pathogenesis to precision diagnosis and treatment

**DOI:** 10.3389/fimmu.2025.1668356

**Published:** 2025-12-12

**Authors:** Lei Li, Fei Ren, Zhuo Liu, Xiaojun Tian, Hongxian Zhang, Guoliang Wang, Shudong Zhang, Lulin Ma

**Affiliations:** 1 Department of Urology, Peking University Third Hospital, Beijing, China; 2 Department of Hernia and Abdominal Wall Surgery, Peking University People’s Hospital, Beijing, China

**Keywords:** kidney transplantation, urothelial carcinoma, pathogenic characteristics, diagnosis, treatment strategies

## Abstract

Kidney transplant recipients (KTRs) exhibit a higher incidence of neoplasms compared to the general population, primarily due to the prolonged administration of immunosuppressive agents and viral infections. In China, the primary type of tumor among KTRs is urothelial carcinoma (UC), which lacks specific clinical manifestations. Accurate diagnosis necessitates the integration of multiple diagnostic modalities, while therapeutic approaches must judiciously balance oncological control with the preservation of renal function, thereby presenting a considerable challenge to the health of KTRs. This article provides a comprehensive review of the epidemiological characteristics, risk factors, diagnostic methodologies, and therapeutic strategies associated with urothelial carcinoma post kidney transplantation (KT), aiming to enhance healthcare professionals’ understanding of this condition and improve patient management.

## Introduction

KT constitutes a highly efficacious intervention for end-stage renal disease (ESRD). Advancements in technology, coupled with the extensive application of immunosuppressive agents, have markedly enhanced both the quality of life and long-term survival rates of recipients. Nonetheless, there has been a concomitant rise in tumor incidence, with the overall occurrence of malignant neoplasms in KTRs being two to three times greater than that observed in the general population (1, 2), which has become one of the major causes of death in KTRs after surgery (3–5). Among the malignancies observed in KTRs, lymphoma and skin cancer are the most prevalent, with genitourinary system tumors following in frequency (6–8). Recently, the incidence of UC post KT has attracted considerable scholarly attention. Notably, there is variability in the reported incidence rates across various studies. A study conducted in Asia reports that the incidence rates of urinary bladder urothelial carcinoma (UBUC) and upper tract urothelial carcinoma (UTUC) in KTRs are 25.5 times and 129.5 times higher, respectively, compared to the general population (9). Among 2,345 KTRs at a specific center, 20 patients were identified with urogenital cancers, including 13 cases of bladder or ureteral cancer (7). A separate retrospective analysis of 5,920 KTRs revealed that 13 cases (0.2%) were diagnosed with UC, of which 8 were bladder cancer (BC), yielding an incidence rate of 0.13%. This rate is significantly higher than the 0.02% observed in the general population (10). In Chinese KTRs, *de novo* urothelial carcinoma is notably prevalent, representing over 40% of all post-transplant malignant tumors (11–13). Presently, there is a limited understanding of the incidence characteristics, risk factors, and treatment strategies for UC following KT. This article seeks to systematically summarize the relevant literature to provide theoretical references and guidance for clinical practitioners.

## Pathogenic characteristics

The majority of UC following KT have been documented to originate from the native urinary tract, but UC can also develop from the allograft (14, 15). Hematuria is a prevalent symptom; however, in KT patients, the diagnosis of tumors may be delayed compared to non-KT patients presenting with hematuria. A comparative study of UTUC patients in KT and non-KT cohorts revealed that the incidence rates of gross and microscopic hematuria in the KT group were 43% and 64%, respectively, which were lower than the corresponding rates of 76% and 86% observed in the non-KT group. Additionally, KT patients exhibiting hematuria demonstrated a higher incidence of non-organ-confined tumors (16). Wu MJ et al.’s study found that the most common initial symptoms in patients were painless gross hematuria and chronic urinary tract infection (UTI), often with bladder irritation symptoms like frequent urination, urgency, and dysuria (17). It is worth noting that abnormal urine cytology may also serve as the initial manifestation of UC in KTRs, accounting for 47.8% of cases (18). However, Wu MJ et al. observed that among patients who had undergone a minimum of three urine cytology examinations before biopsy, only 7 cases (23.3%) were strongly suspected of UC, indicating a high false-negative rate for urine cytology examinations (16). Furthermore, certain patients may present with symptoms associated with urinary tract obstruction, such as hydronephrosis resulting from ureteral tumors. This can lead to clinical manifestations including flank pain, oliguria, anuria, and compromised renal function, all of which significantly jeopardize the patients’ quality of life and prognosis.

In KTRs, UC is frequently high-stage, high-grade, and aggressive. Research indicates that 60% of UC cases in KTRs reach stage pT2 or higher, with 83% being high-grade (19). Compared to non-transplant patients, a higher percentage of female transplant recipients have UTUC staging above pT2 (20). Pathological types may include squamous differentiation and carcinoma sarcomatodes ingredients (21). In KTRs, those with UTUC who test positive for the polyomavirus large T antigen (LTAg) tend to develop the disease earlier (7). UC linked to BK polyomavirus (BKPyV) shows distinct pathological traits, including Glandular differentiation and micropapillary structures, with higher immunohistochemical positivity for LTAg, p53, and p16. Micropapillary UC, a rare and aggressive subtype, makes up 0.6%–1% of cases in the general population but accounts for 60% of cases in KTRs with BKPyV history. It features small clusters of tumor cells surrounded by lacunae, often with lymphovascular invasion and carcinoma in situ (22).

In Chinese KTRs, UC predominantly manifests as multifocal and bilateral. Notably, patients who utilize traditional Chinese medicine exhibit a significantly elevated risk of developing UC, with a higher prevalence observed among female patients (23–25). The onset of UC generally occurs within the first six years following transplantation, which is earlier compared to some Western countries. This discrepancy may be attributed to the frequency of post-transplant monitoring (26, 27). Furthermore, the incidence of UC in the upper urinary tract is greater than in the bladder among Chinese KTRs, contrasting with the pattern observed in dialysis patients (12, 28). In Western countries, UC in RTRs is predominantly bladder cancer, which stands in sharp contrast to the situation in China (23). Due to their immunosuppressed state, KTRs experience faster progression and widespread recurrence of UC compared to the general population. Studies have shown that the five-year recurrence rate following transurethral resection of bladder tumor (TURBT) is 77.7% in KTRs, and 38% in non-kidney transplant patients (29).

## Risk factors

**Figure 1:**
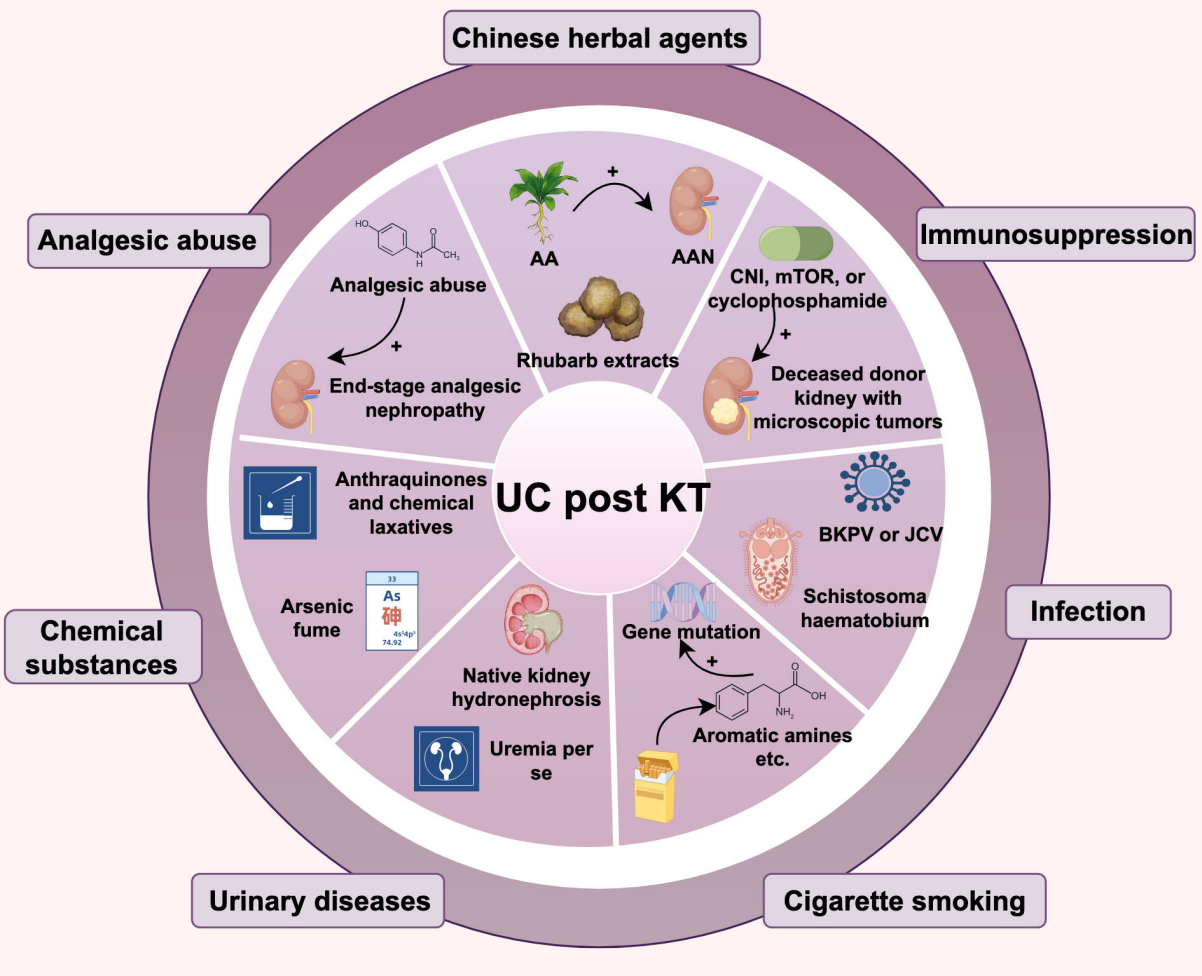
Risk factors for urothelial carcinoma in kidney transplant recipients.

There are various risk factors for KTRs developing UC, including the use of traditional Chinese medicine, immunosuppression, and viral infections, etc. As shown in **Figure 1**.

### Chinese herbal agents

Aristolochic acid (AA) is a potent human nephrotoxin and carcinogen that plays a key role in the pathogenesis of renal fibrosis and UC, often found in traditional Chinese medicine for digestion and weight loss. It’s also used in ESRD patients to reduce serum creatinine (30). AA exposure may explain the higher rates of UC post-transplant in the Chinese population (25, 31), especially among KTRs with a history of Aristolochic Acid Nephropathy (AAN), who face a greater risk of primary kidney UTUC (32). Traditional Chinese medicines with AA, like Isotrema manshuriense (Kom.) H. Huber, may raise UC risk in KTRs. KTRs should be cautious about herbal ingredients’ impact on kidney function and cancer risk, particularly those with AA. In addition, Rhubarb extracts are also key components in certain traditional formulations, like uremic clearance granules. It is mainly used for renal dysfunction or chronic kidney disease, and may also cause UC post KT. Thus, overlooking other Chinese herbal products is unwise, as they too can have serious effects (23, 33), as shown in **Table 1**. In western countries, the etiological factor of Chinese herbal medicine intake is rarely mentioned. Instead, the common etiological factors for UC after renal transplantation in these countries are more closely associated with the use of immunosuppressants, abuse of analgesics, and other related factors. The impact of Chinese herbal medicine intake on the development of this cancer is far less significant than that in China (23).

**Table 1:** High-risk traditional chinese medicine related to kidney injury and UC in KTRs.

Category of traditional Chinese medicine	Key components/related drugs	Mechanisms of kidney injury/UC risk	Clinical implications
AA-containing herbs	AA; Representative drug: Isotrema manshuriense (Kom.) H. Huber	AA is a potent nephrotoxin and carcinogen, inducing renal fibrosis and DNA mutations. It is closely associated with the pathogenesis of UC, especially UTUC in KTRs with a history of AAN	KTRs should avoid using such herbs, especially those with a history of AAN. AA exposure is a key risk factor for UC in Chinese KTRs
Rhubarb extract-containing preparations	Rhubarb extract; Representative drug: Uremic Clearance Granules	Used for renal dysfunction or chronic kidney disease, but long-term use may increase the risk of UC post-KT	Caution is required when using such preparations in KTRs, and regular monitoring for UC is recommended

### Immunosuppression

Evidence indicates that immunosuppressive therapy significantly raises cancer risk in KTRs (34). While these agents prevent kidney rejection, they also impair immune surveillance, allowing tumor cells to evade detectionand and clearance by the immune system, thus increasing UC risk (35). Additionally, long-term use of calcineurin inhibitors (CNIs) like cyclosporine and tacrolimus, as well as mammalian target of rapamycin (mTOR) inhibitors can directly damage DNA, further elevating UC risk (36). However, the lack of immunosuppressants, particularly sirolimus, is also linked to UC post KT. Lai HY et al. found early UTUC post KT was associated with not using sirolimus, which correlated with improved disease-free survival (DFS). And high levels of 7-(deoxyadenosin-N6-yl)aristolactam I (dA-AL-I) in matched normal tissues suggest AA exposure and could be a predictive and prognostic biomarker for new UTUC cases post KT (37). In addition, donor transmission, like a deceased transplant kidney with hidden microtumors, can lead to cancer growth after immunosuppressive therapy (38). Thus, it’s crucial to balance anti-rejection and anti-cancer treatments.

### Virus infection

**Figure 2:**
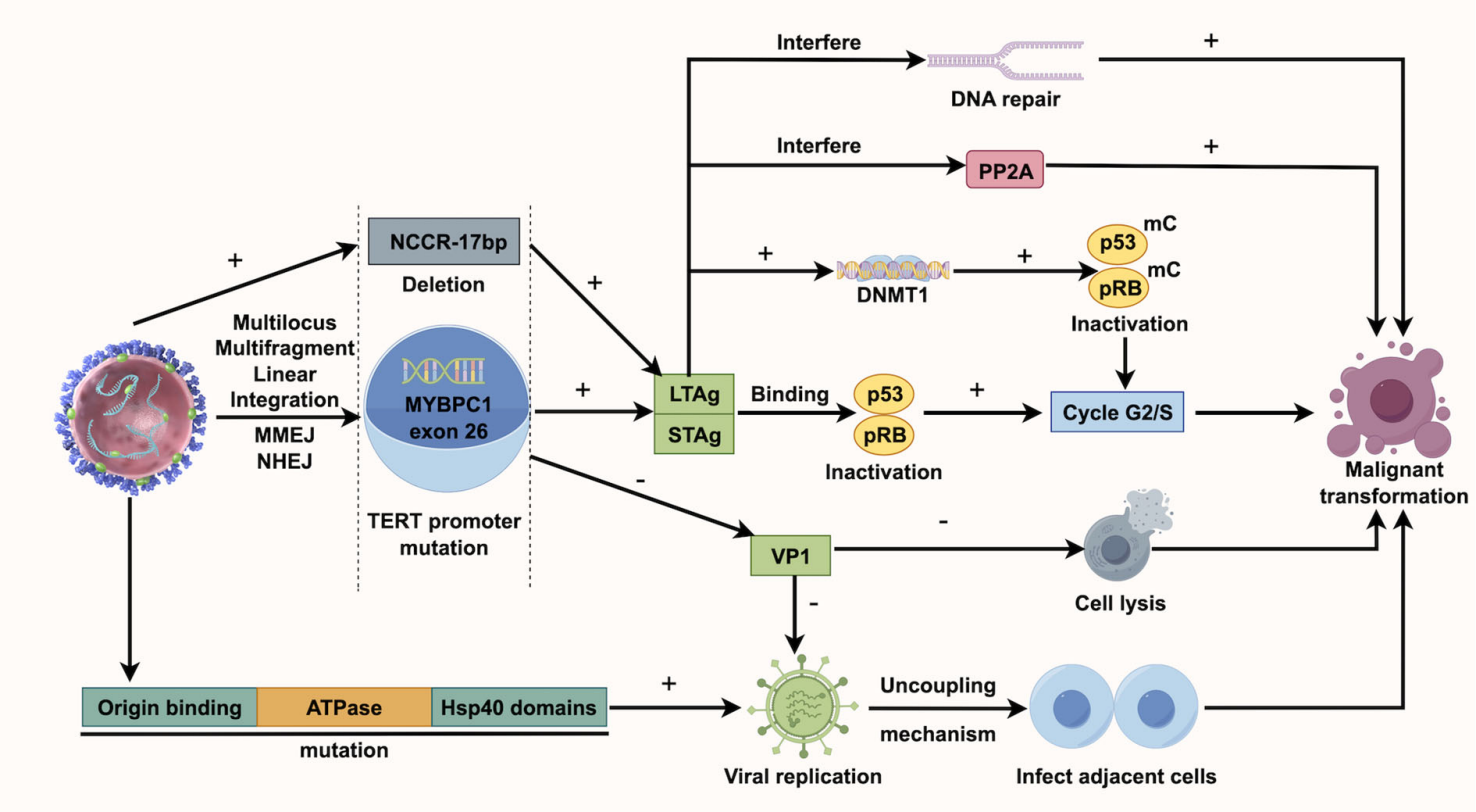
The pathogenesis of BK polyomavirus in urothelial carcinoma post kidney transplantation.

KTRs face a higher risk of viral infections due to immunosuppressive therapy, which may contribute to UC development (39). Human polyomavirus (HPyV), a non-enveloped double-stranded DNA virus, can integrate into the host genome, causing cell cycle disruptions and gene mutations, thereby increasing UC risk (34). BK polyomavirus (BKPV), a common HPyV, usually remains latent in healthy individuals but can reactivate and replicate in KTRs due to prolonged immunosuppressive treatment (40, 41). BKPV’s carcinogenic mechanism may involve its encoded Large T Antigen (LTAg), which can bind to and inactivate the host’s tumor suppressor genes p53 and pRB, resulting in unchecked cell cycle progression and tumor development (42). Chu YH et al. discovered that TAg-positive UC exhibited BKPV integration and higher levels of p16 and p53 compared to TAg-negative UC. Additionally, TAg-positive UC was more likely to present at advanced stages (50% T3-T4), associated with lymph node metastasis (50%), and had a higher UC-specific mortality rate (50%) (43). Kenan DJ et al. discovered that in KTRs with high-grade UC, BKPV disrupts VP1 protein expression and viral replication, meanwhile, deletions occur in the non-coding control region (NCCR). They suggest BKPV contributes to UC by disrupting cell cycle regulation and enhancing genetic instability (44). According to Yan L et al., all instances of polyomavirus-positive UC appeared more than 9 years following transplantation, indicating that the ‘time lapse’ could significantly influence polyomavirus-associated UC and calls for continuous observation (7). Jin Y et al. studied BKPyV integration in the bladder cancer of KTRs using whole-genome and viral capture sequencing. They identified a unique multi-site, multi-fragment linear integration pattern, unlike earlier models, possibly involving microhomology end joining (MMEJ) and nonhomologous end joining (NHEJ). The number of integration sites might correlate with tumor invasiveness, suggesting that monitoring viral load could help prevent related cancers (45). **Figure 2** illustrates the BKPV’s mechanism of action. Besides, John cunningham virus (JCV) is believed to be linked to UC. While its carcinogenic role in the general population isn’t fully established, case reports in KTRs suggest JCV’s involvement in UC, as JCV DNA is frequently found in tumor tissues and is closely associated with tumor development (46). It should be noted that much of the current evidence on the roles of BKPV and JCV in carcinogenesis is primarily drawn from studies of bladder cancer rather than urothelial carcinoma more broadly.

### Toxin and native kidney hydronephrosis

ESRD patients’ urine contains toxins that can lead to UC (47). Most ESRD patients, whether or not they have had a kidney transplant, experience decreased urine output, leading to prolonged retention of metabolic toxins such as aromatic amines and polycyclic aromatic hydrocarbons, and inflammatory mediators in the bladder mucosa. This persistent exposure induces chronic oxidative stress and DNA damage in urothelial cells, creating a pro-carcinogenic microenvironment. Additionally, anuria reduces the elimination of potential carcinogens, such as aristolochic acid metabolites and viral particles that may accumulate in the bladder tissue over time. The bladder, positioned downstream in the urinary tract, has a larger surface area than the upper tract. KT could increase urine flow flushes the bladder. Ho CJ et al. propose this might partly account for the higher UC incidence in KTRs, with UTUC being more common than UBUC (19). Hydronephrosis is also linked to UC development due to urine stasis, recurrent UTI, chronic inflammation of the urinary tract lining, and toxin buildup, resulting in a toxic local environment. Ho CJ et al.’s study confirmed that native kidney hydronephrosis is a remarkably strong independent predictor for post-KT UTUC, with an Odds Ratio (OR) of 35.32 (95% CI, 17.99–69.36; p < 0.001) (19). Among 67 KTRs with UTUC, the incidence of hydronephrosis was 68.7%, significantly higher than the 4.8% in those without UTUC (19). Ho, C.J. et al. discovered that patients with new-onset hydronephrosis post KT had a higher likelihood of developing UC, particularly UBUC. Notably, synchronous UTUC was observed significantly more often in the NKH group (65.2% vs. 21.1%; p = 0.004), which reflects the highly aggressive and multifocal pan-urothelial field change in this patient population. This synergy further underscores the clinical significance of NKH as a key radiological marker for UC screening (48). Thus, diligent monitoring and prompt intervention are crucial for these patients.

### Cigarette smoking

UC is also associated with smoking. In the general population, smoking is one of the primary risk factors for bladder cancer, with approximately 50% of bladder cancer cases linked to smoking (49). Harmful substances from smoking, like aromatic amines and polycyclic aromatic hydrocarbons, enter the bloodstream, reach the bladder, and can cause DNA mutations, leading to cancer. In KTRs, smoking significantly affects bladder cancer risk. Liu S et al. found smoking to be an independent risk factor for bladder cancer post KT. Their analysis showed that smoking raises bladder cancer risk 6.1 times in patients with polyomavirus, while polyomavirus replication in smokers increases the risk 9.7 times, indicating a potential synergistic effect (50).

### Other factors

Besides, risk factors such as exposure to arsenic fumes, analgesic abuse, and chronic inflammatory status in KTRs contribute to the predominance of UTUC among female patients (51–53). Uremia per se have been reported to be predisposing factors for invasive bladder cancer in RTRs (54). Studies have indicated that using anthranoids and chemical laxatives for a year is significantly linked to UTUC risk in RTRs (55). An Egyptian report found that 0.4% of 1865 kidney transplant patients had bladder cancer, often linked to prior schistosomiasis infection (56). Genitourinary system diseases, such as reflux or obstructive uropathy, as well as congenital anomalies (accounting for approximately 40%), are also closely related to the occurrence of UC in RTRs (57).

## Diagnosis strategies

### Imaging examination

Ultrasound is a convenient and reproducible method for detecting kidney and ureter abnormalities, including hydronephrosis and ureteral masses, and is useful for initial UC screening in KTRs (58). Notably, given that Native Kidney Hydronephrosis (NKH) has been validated as an extremely strong independent predictor of post-KT UTUC (19), ultrasound is not only suitable for initial UC screening but also serves as the most effective non-invasive, high-yield method for periodic NKH monitoring in KTRs. This allows for timely identification of NKH, a key radiological marker that signals an elevated risk of UC development. Computed Tomography (CT) offers a clear view of a tumor’s location, size, shape, and relation to nearby tissues, aiding in staging and treatment planning, especially for KTRs suspected of UC. They can confirm lymph node metastasis (59). Retrograde pyelography can detect filling defects in the renal pelvis and ureter, useful for diagnosing UTUC. Each imaging method has its pros and cons, so combining multiple techniques is common in clinical practice to enhance diagnostic accuracy.

### Cystoscopy and biopsy

Cystoscopy is essential for diagnosing bladder cancer by allowing direct bladder lesion visualization and tissue sampling for biopsy. Ureteroscopy with biopsy is used for diagnosing tumors in the renal pelvis and ureter. RTRs with AAN history should have regular cystoscopies (60). However, atrophy and upper urinary tract strictures complicate UTUC diagnosis, often making lesion access and biopsy difficult.

### Urinalysis

Urinalysis can identify hematuria, but its occurrence is much lower in the KT group compared to the non-KT group. Relying solely on hematuria for screening may delay diagnosis, so it is recommended to combine it with imaging methods like ultrasound and CT for early detection of UTUC post KT (16). Although urinalysis can detect cancer cells, its sensitivity is low, especially due to reduced urine volume from ESRD, which limits detection mainly to bladder lesions. The presence of decoy cells and highly atypical cells in urine suggests potential malignancy, warranting further confirmation through immunohistochemistry, such as SV40. Odetola, O.E., et al. found that 17 of 36 cases had urine cytology tests, with 11 positive results and 5 showing both decoy and malignant cells (61). The effectiveness of urine cytology for detecting UTUC is debated (19), but washed urine cytology is crucial in standard UTUC examinations. Bilateral upper tract washing cytology is particularly useful for excluding high-grade UTUC in patients with repeated uncertain urine cytology and negative cystoscopy results (62).

## Management of UC in KTRs

Surgical treatment is the standard for UC in KTRs, but long-term immunosuppression reduces their surgical tolerance, impairs wound healing, and increases infection risk, potentially worsening organ damage. Surgery can also trigger harmful inflammatory responses. There are no universal guidelines for post-transplant UC management, so treatment should be individualized based on tumor characteristics.

### Surgical treatment of UTUC​

Advanced, multifocal, or bilateral UTUC lesions have a poor prognosis, with radical nephroureterectomy (RNU) and excision of cuff of bladder as standard treatments in KTRs (63). Both open nephroureterectomy and laparoscopic nephroureterectomy (LNUT) have similar operative times, but the laparoscopic approach results in less blood loss, shorter hospital stays, and better oncological outcomes (64). However, the use of LNUT in KTRs with UTUC is limited, and there is no consensus on whether open or laparoscopic surgery should be used for patients with UTUC post KT. Wu JT et al. performed LNUT on 11 patients with *in situ* UTUC post KT, reporting no intraoperative complications, an average hospital stay of 6.7 days, stable renal function, and minimal disease progression. They concluded that LNUT is safe and effective, offering benefits like minimal trauma, quick recovery, and acceptable oncological outcomes. LNUT can be executed through either a retroperitoneal or transperitoneal method. The retroperitoneal laparoscopic technique efficiently reveals the renal pedicle, necessitating less dissection and minimizing the risk of injury to intraperitoneal organs. The primary drawback of this method is the restricted working area due to the heightened risk of harming the nearby transplanted kidney (65). Wu JT et al. noted that the laparoscopic method offers great access to the kidney and renal hilum and can be easily switched to open distal ureterectomy to protect the transplanted kidney from damage due to severe adhesions from distal ureteral cancer. They also routinely use this approach for lymph node removal (66). While LNUT offers several benefits, its use in treating UTUC in KTRs is challenging, particularly when the primary tumor and transplanted kidney are on the same side, as limited space heightens the risk of damaging the transplant (65, 66). Chang NW et al. note that despite the challenges of open surgery for KTRs, including scar tissue around the graft, and difficult ureteral dissection in laparoscopic procedures, in their team, the proportion of open surgery was higher in the KT group than in the non-KT group (67). Despite limited randomized trials, numerous case reports suggest that transplant recipients gain from laparoscopic surgery, experiencing less pain, shorter hospital stays, quicker recovery, and fewer wound complications (68). The key concern is the long-term outcomes of LNUT for these patients, but studies are scarce. Long-term follow-up is essential to evaluate LNUT’s effectiveness in treating UTUC in KTRs.

There is ongoing debate about performing prophylactic contralateral RNU for KTRs with unilateral UTUC, as studies indicate KTRs have a high risk of developing synchronous bilateral UC (17, 51). Furthermore, recurrence on the opposite side after a unilateral radical nephroureterectomy (URNU) is common. According to Fang et al, patients who underwent transplantation had a more than 15 times higher risk of developing tumors on the opposite side compared to those who did not undergo transplantation, with 60% of post-renal transplant patients developing contralateral tumors (69). The study by Huang et al. found that among patients who had renal transplants or were on regular dialysis, the contralateral recurrence rate over five years was 38.3%, with every recurrence occurring within the first three years (70). Therefor, they recommend simultaneous bilateral radical nephroureterectomy (SBRNU) once one side is diagnosed (69, 70). Zhang Q et al. studied the effectiveness of SBRNU versus URNU in treating newly diagnosed UTUC post KT, involving 48 patients (21 SBRNU, 27 URNU). SBRNU resulted in longer surgeries and hospital stays, but similar blood loss and perioperative complications compared to URNU. At a 65-month follow-up, SBRNU showed better progression free survival (PFS) and cancer specific survival (CSS). The author suggests that SBRNU can enhance survival rates without impacting perioperative outcomes. It may be a suitable treatment for high-risk patients, particularly females with prolonged AA exposure, prone to bilateral UTUC post KT (71). Lin KJ et al. studied 44 cases of KTRs with UTUC, finding that PFS was significantly better in the SBRNU group than in the URNU group (72). Kao YL et al. observed that 41% of UTUC cases post KT were synchronous and recommended prophylactic SBRNU due to high contralateral recurrence rates and lack of early screening methods. However, given the 0.2%–2.63% incidence of UTUC post KT, they advised against routine prophylactic SBRNU for all patients, suggesting instead close postoperative monitoring. SBRNU with bladder cuff resection is strongly recommended if UTUC is suspected or if there is high risk, while considering patient preferences (51). While this method reduces the risk of postoperative urinary tract infections and upper urinary tract tumor recurrence, it may raise the risk of other complications due to reduced residual urinary tract (56). Lang, H et al. performed systemic SBRNU on four patients but did not find any tumors (73). Therefore, some authors suggest that SBRNU heightens health risks for KTRs, recommending URNU first, with subsequent careful monitoring and evaluation (66). In summary, treatment for KTRs with unilateral UTUC should be personalized. The benefits of prophylactic SBRNU are uncertain, so it shouldn’t be standard practice. It’s important to weigh the risk of postoperative infections or tumor recurrence in the upper urinary tract against the advantages of maintaining some urinary function. For patients with AAN at high risk of synchronous UTUC, decisions should be made after thoroughly evaluating renal function, tumor traits, and surgical tolerance.

### Surgical treatment of UBUC

Muscle invasive bladder cancer (MIBC) occurs in 37% of KTRs, a higher rate than in the general population, with most cases diagnosed at advanced stages (74, 75). Treating bladder cancer in KTRs is complex due to the need to balance effective cancer treatment with maintaining kidney function and proper urinary drainage. Due to the aggressive nature of UC in KTRs, a conservative endoscopic approach should not be applied to the KT population. While transurethral resection of bladder tumor (TURBT) appears reasonably safe for low-risk diseases in the general population (76). MIBC is generally more aggressive than non-muscle invasive bladder cancer (NMIBC), with a higher progression rate, poorer prognosis, and lower survival rate (77). Therefore, early radical cystectomy (RC) and pelvic lymph node dissection (PLND) are widely recommended (78). Comparative data from a series of cystectomies indicate that extended lymph node dissection improves overall survival (79), though some researchers prefer to avoid it on the transplant side to prevent blood supply damage. If needed, the opposite side’s dissection can be extended (77). When a cystectomy is needed in patients who have undergone a kidney transplant, the choice of urinary diversion must be evaluated and adjusted according to the renal transplant’s performance. Reconstruction options vary from basic cutaneous ureterostomy to an orthotopic neobladder, with the latter advised only for patients whose glomerular filtration rate is stable at 50 ml or higher. In the past, ileal conduits or Kock pouches were used for renal transplant recipients needing cystectomy, despite the high risk of renal infection and graft deterioration in immunosuppressed patients (77). Additionally, urinary diversion during cystectomy is crucial, with the studer *in situ* bladder procedure being a popular choice after RC. This technique uses an ileum segment to create a urinary reservoir, connecting the ureter to the urethra, allowing patients to regain near-normal urinary function. It’s suitable for those with MIBC or high-risk NMIBC, requiring careful evaluation of intestinal function and urethral sphincter status. Successful cases include KTRs undergoing this procedure. Manassero F et al. assert that the studer technique is effective for urinary tract reconstruction in bladder cancer patients post KT due to its adaptability to short ureters and its ability to prevent reflux (77, 80). However, when selecting a urinary diversion method, consider patient comfort and complication risks. While orthotopic neobladder is more physiologically suitable, it can heighten urinary tract infection risk from intermittent catheterization and cause metabolic issues due to urine absorption by the intestines. Yavuzsan AH et al. conducted a RC with ileal conduit diversion on a young female with invasive bladder cancer, featuring sarcomatoid and squamous cell variants, three years post KT. The procedure was likely chosen for its simplicity and lower complication risk, crucial for preserving the transplanted kidney and the patient’s overall health (81). In complex cases with both upper and lower urinary tract lesions, advanced surgeries like laparoscopic bilateral nephroureterectomy with PLND and orthotopic neobladder may be necessary (82).

### Adjuvant therapy

UC is often multifocal, and adjuvant chemotherapy is crucial for advanced or high-risk cases. Adjuvant chemotherapy plays a significant role in the comprehensive treatment of UC post KT. However, immunosuppressed patients may poorly tolerate chemotherapy and face more complications, requiring careful patient selection. While platinum-based chemotherapy is the primary treatment for advanced UC, its nephrotoxicity limits its use in KTRs (83). Chang NW et al. analyzed 57 patients with advanced UTUC (stage T2 or higher) who underwent nephroureterectomy and bladder cuff excision, followed by adjuvant chemotherapy with gemcitabine plus cisplatin or carboplatin. The study included 23 KTRs and 34 non-transplant patients. Results indicated the five-year disease-free survival (DFS) and overall survival (OS) rates were 45.7% vs. 70.2% and 62.8% vs. 77.6%, for the KT and non-KT groups. Hematologic toxicities showed significant differences between the KT and non-KT group. The KT group had more severe neutropenia, anemia, and thrombocytopenia compared to the non-KT group. In contrast, non-hematologic toxicities, including nausea/vomiting, nephrotoxicity, hepatotoxicity, and skin rash, did not differ statistically between the two groups. Notably, only 3 patients in the KT group and 2 in the non-KT group developed >grade 2 nephrotoxicity, which was reversible after treatment, indicating mild and acceptable impacts of chemotherapy on graft kidney function; additionally, recombinant human granulocyte colony stimulating factor was used to manage high-grade hematologic toxicities, though nearly half of KT patients and one-third of non-KT patients still required chemotherapy dose reductions. These findings are valuable for clinical treatment guidance (67). Additionally, Zhang, P., et al. found that gemcitabine and cisplatin (GC) chemotherapy was somewhat effective for locally advanced disease. In seven KTRs with advanced UC, pre- and postoperative GC treatment resulted in one complete response, two partial responses, and four stable cases, with a 43% overall efficacy rate. Myelosuppression was the primary toxicity and side effect linked to the GC regimen. Other adverse reactions included reversible nephrotoxicity, gastrointestinal and cutaneous manifestations, and phlebitis. Hematologic toxicities, meanwhile, encompassed reversible leukopenia, thrombocytopenia, and anemia (84). Wang, Z.P. et al. studied 22 patients with advanced UC post KT. Eleven received surgery plus adjuvant chemotherapy (GC regimen), and the other eleven had surgery only. The group receiving adjuvant chemotherapy showed significantly better survival. KTRs with advanced UC who received surgery plus adjuvant GC chemotherapy had significantly better OS than those who only underwent surgery, with the median OS extended by 17 months. Hematologic toxicities occurred at a relatively high incidence, leading to dose reduction of chemotherapeutic agents in 45.5% of patients. Among nonhematologic toxicities, gastrointestinal reactions were the most prevalent. Grade 1 nephrotoxicity was documented in 3 patients, with no cases of higher-grade nephrotoxicity observed. Notably, serum creatinine and blood urea nitrogen levels did not show significant changes during chemotherapy. Of these eleven patients, seven had UTUC (85). These data confirm that platinum-based AC significantly improves disease control and survival in KTRs with advanced UC. While Wang, Z.P. et al. confirmed the safety of platinum-based chemotherapy for KTRs, its long-term effects on patients with ≥T2 disease are uncertain. Du et al. reported no significant impact of this chemotherapy on the prognosis of such patients. Furthermore, the effectiveness of the GC or Gemcitabine and Carboplatin (GCa) regimen in preventing distant metastasis in these recipients remains unproven (83).

Bladder instillation chemotherapy effectively reduces NMIBC recurrence. Despite lacking evidence for benefits in KTRs, immediate postoperative instillation is advised due to high recurrence risk (78). Elkentaoui H et al. conducted TURBT on NMIBC patients post KT, followed by at least one mitomycin C instillation, observing no mitomycin C-related complications. Neuzillet et al. concluded that mitomycin C is safe for KTRs and may be beneficial without added risk (86, 87). For patients with tumor recurrence after intravesical mitomycin C therapy, intravesical gemcitabine may be an option. Although typically given intravenously for metastatic bladder cancer, studies indicate that a 1000 mg intravesical dose can help prevent recurrence in high-grade NMIBC patients unresponsive to BCG therapy (88). BCG is an effective treatment for high-risk NMIBC, typically requiring at least a year of maintenance to lower recurrence and progression rates (54). However, its use in KTRs is debated due to safety concerns in immunosuppressed patients. First, the effectiveness of BCG depends on the patient’s immune response, and it may be less effective in those with weakened immunity. However, studies have shown that BCG bladder instillation chemotherapy is both safe and effective (89, 90). Second, due to the patient’s impaired immune response, BCG may spread systemically, leading to significant side effects. There have been case reports of systemic infection following bladder instillation of BCG (91). Currently, only a limited number of medical centers have experience with BCG in KTRs, and even fewer have enough patients to create guidelines. Future research should focus on optimizing BCG treatment for KTRs (92).

Neoadjuvant or adjuvant immunotherapy currently improves survival in advanced UC, especially for patients ineligible for cisplatin with few other options (93). Immune checkpoint inhibitors (ICIs), which enhance the immune response against tumors by blocking PD-1/PD-L1 pathways, have proven effective and safe in treating platinum-resistant metastatic UC. Various ICIs have demonstrated efficacy and safety comparable to chemotherapy, and pembrolizumab outperformed chemotherapy in a phase III trial (94). However, immunotherapy for UC in KTRs may still be a double-edged sword, as it may trigger T cells to attack both tumor and donor antigens. Research indicates that PD-1 can lead to kidney transplant rejection in some cases and often accelerates tumor growth (95). Some KTRs have effectively managed metastatic UC with anti-PD-1 monotherapy. In this instance, the patient showed partial tumor regression without any transplant rejection during the treatment (96). Wu CK et al. reported on a kidney transplant patient with metastatic UC treated with pembrolizumab, bevacizumab, cisplatin and gemcitabine with a continued immunosuppressants (mycophenolate mofetil and tacrolimus), leading to notable tumor reduction and stable graft function (97). Therefore, ICIs can potentially manage cancer without causing rejection. However, using ICIs in KTRs demands careful balancing of immune activation and rejection risk. As an antibody-drug conjugate (ADC), Enfortumab ventodin (EV) specifically targets the Nectin-4 protein. This protein is highly expressed in UC and has demonstrated significant efficacy in patients who have failed platinum-based chemotherapy and ICIs therapy (98). Studies have shown that EV is also well-tolerated in patients with renal impairment (99), which is particularly important for kidney transplant recipients. However, the safety and efficacy of EV in kidney transplant patients require further investigation. New treatments, like ADCs and ICIs combination therapies, are being studied and may improve patient outcomes (100). Fibroblast growth factor receptor (FGFR) inhibitors, such as erdafitinib, have shown some success in treating UC, but more experience is needed for use in post-KT patients.

### Adjustment of immunosuppressants

When a kidney transplant recipient is diagnosed with urothelial carcinoma, it’s crucial to reduce or adjust immunosuppressive drugs to boost immune surveillance. This adjustment is necessary, especially when using ICIs, to balance tumor treatment and prevent transplant rejection (59). Close monitoring of kidney function is essential to avoid rejection, making the careful adjustment of the immunosuppressive regimen a key part of treatment.

Tacrolimus, a CNI drug, has advanced transplantation with strong short-term outcomes, but its chronic nephrotoxicity poses a challenge (101). Similarly, cyclosporine, another CNI drug, can lead to malignant tumors (102). Conversely, mTOR inhibitors may lower the risk of cancers like non-melanoma skin cancer and Kaposi’s sarcoma (103). For KTRs with newly diagnosed UC, mTOR inhibitors are typically favored over CNI drugs. Some experts recommend minimizing CNI use in immunosuppressive regimens for those at high risk of tumor development (9). Combining CNI and mTOR inhibitors allows for lower CNI doses, minimizing adverse reactions without compromising transplant outcomes; rejection rates remain similar to standard doses, while renal function improves.

Hu XP et al. assessed the safety and effectiveness of combining rapamycin (RPM) with low-dose CNI in 15 KTRs with UC. The regimen replaced mycophenolate mofetil (MMF) or azathioprine (Aza) with RPM, adjusting doses to maintain 4–6 μg/L blood levels, and reduced CNI doses to one-third after stabilization. All patients received surgical treatment and intravesical chemotherapy. Over two years, 9 patients had no tumor recurrence, 2 had two recurrences, and 4 had one. No acute rejection occurred, with hyperlipidemia and thrombocytopenia as common side effects. This approach appears to inhibit tumor growth, enhance graft function, and reduce cyclosporine nephrotoxicity, showing promising safety and efficacy (104). Combining mTOR inhibitors with tacrolimus regimens lowers the risk of UC post KT (105). However, Besarani et al. found that altering or stopping immunosuppressive therapy alongside chemotherapy offers limited benefits for patients with metastatic disease post KT (106).

In summary, adjusting immunosuppression regimens for post-KT patients with urothelial carcinoma requires careful monitoring of tumor recurrence, kidney function, and adverse reactions. Treatment should be personalized based on tumor status, incorporating surgical resection and local infusion chemotherapy to improve efficacy and quality of life. This complex process necessitates further clinical research to optimize outcomes. As shown in **Table 2**.

**Table 2:** High-risk immunomodulatory agents related to kidney injury and UC in KTRs.

Class of immunomodulatory agents	Representative drugs	Mechanisms of kidney injury/UC risk	Clinical implications
Calcineurin Inhibitors (CNIs)	Cyclosporine, Tacrolimus	Impair immune surveillance, enabling tumor cell escape;Directly damage DNA;Chronic nephrotoxicity affects renal function	Long-term use requires strict monitoring of drug concentrations and renal function. Dose adjustment or replacement with other agents may be considered for high-risk patients
Mammalian Target of Rapamycin (mTOR) Inhibitors	Sirolimus, Everolimus	Reduced risk of non-melanoma skin cancer and Kaposi’s sarcoma, but lack of sirolimus is associated with early UTUC post-KT;Combined with low-dose CNIs can reduce CNI-related nephrotoxicity	Preferred over CNIs for KTRs with newly diagnosed UC; dose adjustment should be based on blood concentration monitoring
Other Immunosuppressants	Mycophenolate mofetil (MMF), Azathioprine (Aza)	Indirectly increase UC risk by suppressing immune surveillance	May be replaced with mTOR inhibitors in KTRs diagnosed with UC to enhance anti-tumor immune response

## Prognosis and screening

The prognosis for UC post KT is poor, with high recurrence and mortality rates. A propensity-matched study showed that ESRD patients who received a kidney transplant had worse cancer outcomes for UTUC than those who did not (107). The prognosis of UC post KT is significantly affected by factors such as tumor staging, particularly stages ≥ T2 and positive lymph nodes (N+), which are independent risk factors for cancer-specific mortality. In a study of 106 patients with newly diagnosed UTUC post KT, cancer-specific survival rates were 89.2% at 1 year, 73.2% at 5 years, and 61.6% at 10 years (108). In addition, patient-specific factors, like hydronephrosis in the affected kidney and female gender, significantly influence UTUC prognosis (19). UC linked to polyomavirus infection often occurs earlier, affecting outcomes. Treatment choices also impact prognosis; radical surgery can improve tumor control but may not be suitable for all patients due to their health conditions (29).

Monitoring for recurrence and long-term follow-up are crucial in managing UC post KT. Close attention to changes in patient symptoms is essential. Even though hematuria might be atypical in KTRs, any occurrence of hematuria or flank pain should raise suspicion for tumor recurrence. In addition, regular physical exams, lab tests, and imaging can promptly identify tumor recurrence. Urine cytology is useful for monitoring urothelial carcinoma. In a study of patients with UC post KT, the test showed 82% sensitivity and 97% specificity for detecting recurrence (109). Regular ultrasounds can identify abnormalities in the kidneys, ureters, and bladder. Given the quantified predictive value of NKH for UC, we specifically recommend periodic ultrasound monitoring of the native kidneys every 6–12 months to detect NKH promptly. Once NKH is identified via ultrasound, proactive and comprehensive screening of the entire native urinary tract is imperative. Post-surgery CT urography is useful for detecting recurrent tumors in the upper urinary tract and bladder in UTUC patients. Additionally, routine cystoscopy is crucial for monitoring bladder tumor recurrence. Follow-up schedules should be personalized based on the patient’s condition, with more frequent visits soon after surgery and gradually extended intervals. High-risk patients, like those with advanced tumors or multiple infections, require more intensive follow-ups. Utilizing various monitoring methods aids in early detection of tumor recurrence, ensuring timely treatment. Collaboration among a multidisciplinary team for tailored treatment and vigilant follow-up enhances patient outcomes.

## Conclusion

**Figure 3:**
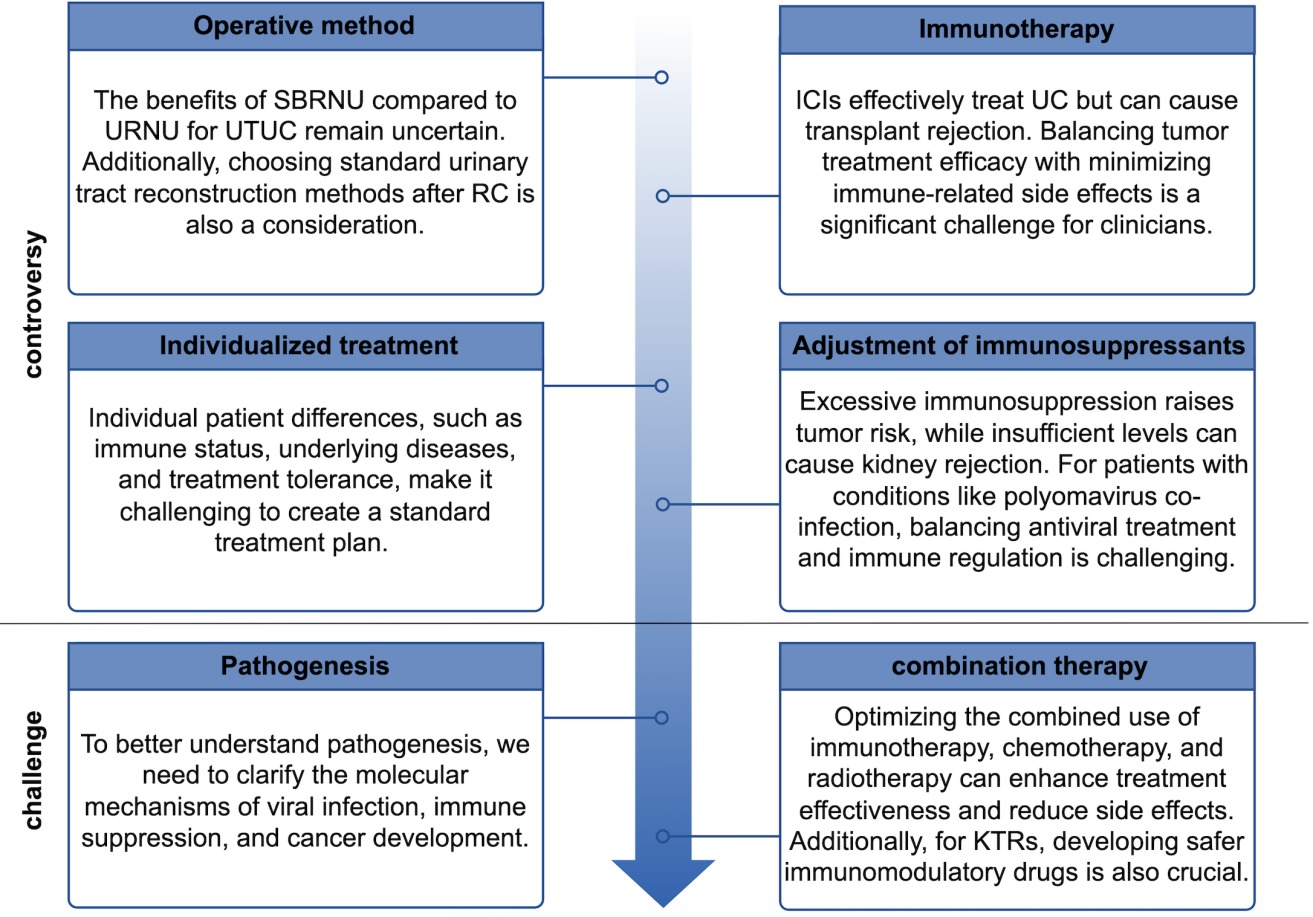
Controversies and challenges faced by kidney transplant recipients with urothelial carcinoma.

The incidence of UC post KT is markedly elevated compared to that in the general population. Within this cohort, female patients with a history of AAN constitute a high-risk group for the development of UC post KT and should be prioritized for screening. During clinical evaluations, particular attention should be directed towards the patient’s native urinary tract. Periodic ultrasound monitoring of native kidney hydronephrosis should be integrated as a core component of post-KT screening protocols, it is recommended that screening commence as soon as possible following KT. Early detection, timely intervention, and regular follow-up are essential for optimizing patient outcomes. While managing the tumor, it is imperative to preserve renal function to the greatest extent possible, thereby enhancing the quality of life and prognosis for patients with UC post KT. Future research should focus on addressing current gaps in the literature. Specifically, prospective multicenter trials are needed to explore in depth the interactions between immunosuppression, UC progression, and emerging therapies in kidney transplant recipients, thereby providing a basis for developing standardized clinical management guidelines. **Figure 3** summarizes the controversies and challenges faced by KTRs.

## Glossary

KT: Kidney transplantation
ESRD: End-stage renal disease
KTRs: Kidney transplant recipients
UC: Urothelial carcinoma
UBUC: Urinary bladder urothelial carcinoma
UTUC: Upper tract urothelial carcinoma
UTI: Urinary tract infection
BKPyV: BK polyomavirus
AA: Aristolochic acid
AAN: Aristolochic acid nephropathy
CNI: Calcineurin inhibitor
mTOR: Mammalian target of rapamycin
DFS: Disease-free survival
HPyV: Human polyomavirus
BKPV: BK polyomavirus
LTAg: Large T antigen
NCCR: Non-coding control region
MMEJ: Microhomology end joining
NHEJ: Nonhomologous end joining
JCV: John Cunningham virus
NKH: Native kidney hydronephrosis
CT: Computed tomography
RNU: Radical nephroureterectomy
LNUT: Laparoscopic nephroureterectomy
URNU: Unilateral radical nephroureterectomy
SBRNU: Simultaneous bilateral radical nephroureterectomy
PFS: Progression-free survival
CSS: Cancer-specific survival
MIBC: Muscle-invasive bladder cancer
NMIBC: Non–muscle-invasive bladder cancer
TURBT: Transurethral resection of bladder tumor
RC: Radical cystectomy
PLND: Pelvic lymph node dissection
OS: Overall survival
GC: Gemcitabine + Cisplatin
GCa: Gemcitabine + Carboplatin
BCG: Bacillus Calmette-Guérin
ICIs: Immune checkpoint inhibitors
ADCs: Antibody–drug conjugates
EV: Enfortumab ventodin
FGFR: Fibroblast growth factor receptor
RPM: Rapamycin
MMF: Mycophenolate mofetil
Aza: Azathioprine
